# Prolonged Ileus after Colorectal Surgery, a Systematic Review

**DOI:** 10.3390/jcm12185769

**Published:** 2023-09-05

**Authors:** Anzil Shereef, David Raftery, Fraser Sneddon, Katy Emslie, Lyn Mair, Craig Mackay, George Ramsay, Patrice Forget

**Affiliations:** 1 Institute of Applied Health Sciences, University of Aberdeen, Foresterhill Campus, Aberdeen AB25 2ZN, UK; 2 NHS Grampian, Foresterhill Campus, Aberdeen AB25 2ZN, UK; david.raftery2@nhs.scot (D.R.); katy.emslie@nhs.scot (K.E.); lyn.mair@nhs.scot (L.M.); craig.mackay@nhs.scot (C.M.); george.ramsay@nhs.scot (G.R.); 3 NHS Highland, Inverness IV2 3BW, UK; fraser.sneddon@nhs.scot; 4 Clinical Chair in Anaesthesia, University of Aberdeen Honorary Consultant, NHS Grampian, Aberdeen AB25 2ZN, UK

**Keywords:** ileus, ileus prevention, prolonged ileus, systematic review, network meta-analysis, colorectal surgery, ileus rates

## Abstract

Background: The development of prolonged post-operative ileus (POI) remains a significant problem in the general surgical patient population. The aetiology of ileus is poorly understood and management options/preventative measures are currently extremely limited. The pathophysiology leading to a post-operative ileus is relatively poorly understood, and there is no validated method to estimate ileus occurrence or duration. Ileus in the post-operative period commonly occurs following major colorectal surgery and leads to painful abdominal distension, vomiting, nutritional deficit, pneumonia, prolonged hospital stays and susceptibility to hospital-acquired infection. An increased hospital stay, the burden of treatment costs and the burden on the health system highlight the importance of future research on finding definitions, preventions and predictions of ileus. Methods: A systematic literature review was performed to identify randomized controlled trials (RCTs) comparing the rate of ileus on various treatments for prolonged post-operative ileus following colorectal surgery. A confidence evaluation in a meta-analysis were performed using CINeMA. Direct and indirect comparisons of all interventions were simultaneously carried out using a network meta-analysis. The level of certainty was appraised using the Grading of Recommendations, Assessment, Development and Evaluations (GRADE) approach. The method of assessing the risk of bias, the quality assessment, used the Cochrane Risk of Bias 2 tool (RoB2). Results: Among the seven included studies, the majority suffered from considerable within-study bias, affecting the confidence rates of study findings. Heterogeneity and incoherence made the pairwise meta-analysis and ranking of interventions unfeasible. Indirect comparisons were considered unreliable due to this incoherence. Conclusions: This systematic review, with a confidence evaluation in the network meta-analysis, determined that there is a knowledge gap in the field of study on prolonged ileus following digestive surgery. The current evidence suffers from heterogeneity and incoherence more than imprecision. There is a gap in the data on ileus occurrence in interventional trials for digestive surgery. This could inform clinicians and trialists to better appraise the current literature and plan future trials.

## 1. Introduction

The development of prolonged post-operative ileus (POI) remains a significant problem in the general surgical patient population. Yet, its multifactorial nature means its etiology is poorly understood and management options/preventative measures are currently extremely limited. A post-operative ileus is defined as the interval from the surgery until the passage of the flatus/stool and the tolerance of an oral diet [1]. However, often after abdominal surgery, a prolonged ileus occurs which leads to an increased length of hospital stay, prolonged recovery time and a substantial strain on resources [2]. The importance of these complications renders this particular situation concerning for the clinician. Prolonged ileus can be defined as abdominal distension, functional occlusion, no oral diet tolerance and vomiting [3]. The pathophysiology leading to a post-operative ileus is relatively poorly understood and there is no validated method to estimate ileus occurrence or duration. Abdominal distension and absolute constipation are the most common symptoms reported, which can cause profound pain in the post-operative patient [2]. The mainstay of managing an ileus is to use the ‘drip and suck’ method: positioning an intravenous cannula to deliver fluids and a nasogastric tube to decompress the bowel while waiting for the bowel motility to return independently. Ileus in the post-operative period commonly occurs following major abdominal surgery and leads to painful abdominal distension, vomiting, nutritional deficit, pneumonia, prolonged hospital stays and susceptibility to hospital-acquired infection [4]. This systematic review is carried out with the aim of analysing the pathophysiological significance of the condition and the severity of the data gap.

A recent investigation of a post-operative ileus in a US colorectal department provided clear evidence of healthcare burden with increased length of stay (11.4 days vs. 5.12 days, *p* < 0.001) and cost of hospitalization (38,000 USD vs. 22,000 USD, *p* < 0.001) for patients that developed an ileus compared to those who did not [5]. Given the reported incidence of post-operative ileus being 10–30% [6,7], elucidating whether specific targeted strategies and factors can be put into place (to decrease ileus frequency or length of symptomatology) could potentially greatly improve patient outcomes and reduce burden on already stretched healthcare resources. Research investigating the definition, prediction and prevention of ileus has now become a highlighted direction of future work within colorectal surgery [8]. In this work, our aim was to adopt a systematic method, to review the literature in the field and to identify, compare and rank interventions which have been tested to decrease the incidence of prolonged post-operative ileus following colorectal surgery.

## 2. Methods

### 2.1. Data Sources

A systematic search was carried out on MEDLINE, Cochrane Central Register of Controlled Trials (CENTRAL), Embase, Web of Science (last update on 12 May 2022) with no restriction for publication period. The search strategy focused only on contexts and terms describing the approaches taken place in the context of ileus combined with a MEDLINE filter for interventions on controlled trials.

### 2.2. Search Strategy

A complete data search was conducted with a specific filter for controlled trials designed in the context of post-operative ileus after major colorectal surgery. The search was performed using appropriate medical terminologies such as ‘ileus’, ‘colorectal surgery’, ‘colon’, ‘colectomy’, ‘colostomy’, ‘ileostomy’ and ‘Inflammatory Bowel Diseases’. The study’s restrictions included no human trials and the language of publications. The systematic review was performed in line with the protocols established in the Preferred Reporting Items for Systematic Reviews and Meta-Analyses (PRISMA) and the Cochrane Handbook for Systematic Reviews of Interventions version 6.1 [9]. The study protocol, with early search strategies and eligibility criteria, was registered on the PROSPERO website on 29 June 2022 (CRD42022341390).

### 2.3. Study Screening

The inclusion and exclusion criteria were planned for the structured and systematic screening of the study data according to the PICO (Population, Intervention, Comparison, Outcomes) methodology by PRISMA guidelines. Population (P): post-operative elective colorectal patients who developed an ileus; Intervention (I): factors that decrease ileus rates; Control (C): control groups used; Outcome (O): primary: ileus (incidence, duration); secondary: length of hospital stay. Exclusion criteria were added for non-human subjects, non-English language studies, trials without ileus rates as the primary outcome, reviews, case reports, conference abstract, letters, non-published trials and ongoing trials.

Rayyan QCRI software version 1 was used by two authors (AS and PF) to screen the study titles and abstracts of the literature based on the inclusion or eligibility criteria. Any conflicts during the screening were discussed and resolved by a third party (co-author).

### 2.4. Data Extraction

Data screening was carried out by two review authors from a full text review of potential eligible studies. Disagreements were disputed by a third reviewer (co-author). A standardised, pre-piloted Excel form was used to extract data from the included studies for an assessment of the study quality and evidence synthesis. The extracted information included: study name, authorship, year, study population, population size, details of the intervention, procedure, primary outcomes, secondary outcomes, complications and information for the assessment of the risk of bias.

### 2.5. Data Synthesis

The risk ratios and risk of bias were analysed and synthesised using the Cochrane RevMan5 software, version 5.4.1 [10]. A meta-analysis for the direct and indirect evidence of each intervention was performed for analysing indirectness, imprecision, heterogeneity, incoherence and confidence rating using CINeMA (version 2.0.0), a confidence meta-analysis software, version 2.0.0 [11]. Due to the limited number of studies per intervention, we did not perform pairwise comparisons (a classical meta-analysis method), calculate the I^2^ statistic or perform a sensitivity analyses. The quantitative heterogeneity between the studies was not measured. Only the clinical estimates based on research quality, demographic and intervention factors were used to determine the heterogeneity.

### 2.6. Risk of Bias Analysis

The risk of bias in the included studies was appraised by considering the following characteristics: *Randomization sequence generation*: was the allocation sequence adequately generated? *Treatment allocation concealment*: was the allocated treatment adequately concealed from study participants and clinicians and other healthcare or research staff at the enrolment stage? *Blinding*: were the personnel assessing outcomes and analysing data sufficiently blinded to the intervention allocation throughout the trial?

*Completeness of outcome data*: were the participant exclusions, attrition and incomplete outcome data adequately addressed in the published report?

*Selective outcome reporting*: was there evidence of selective outcome reporting and might this have affected the study results? Other sources of bias: was the trial apparently free of any other problems that could produce a high risk of bias? Disagreements between the review authors over the risk of bias in particular studies were resolved by discussion, with the involvement of a third review author where necessary. After two reviewers were involved in the quality assessment, with disagreements between the reviewers’ judgements being resolved by a third, the results of the assessment informed the data synthesis. The level of certainty was appraised using the Grading of Recommendations, Assessment, Development and Evaluations (GRADE) approach, a program-based analysis, and the outcome was appraised using Cochrane RevMan5 software [10] and CiNeMA. The study outcome of the risk of bias is summarised in Figure 2.

## 3. Results

### 3.1. Search Results and Study Selection

**Figure 1:**
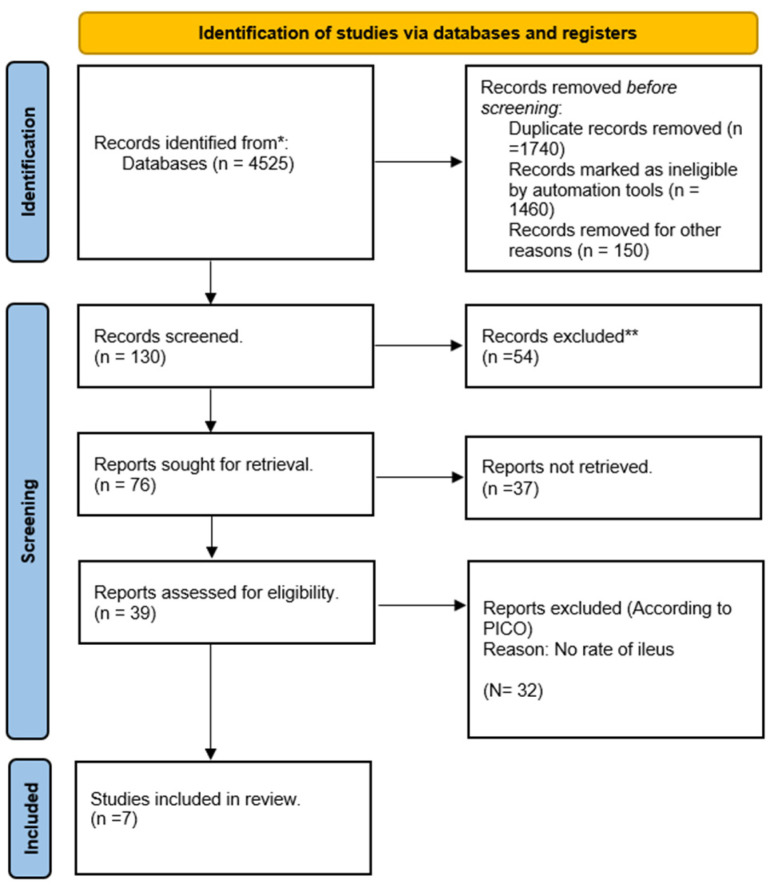
PRISMA diagram showing number of studies included in each stage of the literature search. * Consider, if feasible to do so, reporting the number of records identified from each database or register searched (rather than the total number across all databases/registers). ** If automation tools were used, indicate how many records were excluded by a human and how many were excluded by automation tools.

The PRISMA flow diagram (Figure 1) summarises the search strategy’s outcomes. The initial search results consisted of 749 from MEDLINE, 1922 from Embase, 723 from CENTRAL and 1131 from the Web of Science. From the 4525 studies, we deduplicated and removed 1740 studies using RefWorks Legacy. Screening was performed on 2785 studies initially based on the inclusion criteria, and 67 studies were selected. Among the 67 randomized controlled trials, only 7 papers had documented data on our study’s primary outcome, the rate of ileus. Only studies which exclusively provided data on ileus rates were selected for inclusion. During the screening process, there were no conflicts between reviewers.

### 3.2. Study Characteristics

Data were extracted from seven studies with a clear documentation of the rate of ileus. The study design and characteristics are summarised in Table 1. One trial by Zhang et al. [12] conducted on 302 individuals examined the effects of dexamethasone on the ileus. Another clinical trial included for review by Gomez-Izquierdo et al. [13] studied the effects of goal-directed fluid therapy on the ileus with traditional fluid therapy used as the control. HanGeurts et al. [14] compared the rate of ileus and complications in patients following a free diet versus patients following a conventional diet. Lambrichts et al. [15]’s study focused on the efficiency of nicotine chewing gum over normal chewing gum for the prevention of ileus after colorectal surgeries. Zaghiyan et al. [16]’s study tried to establish the effects of chewing gum on the ileus rate after surgeries, with their control group using no ‘chewing gum’. Peters et al. [17] examined the effects of lipid-enriched enteral nutrition for ileus prevention after colorectal surgeries. Their control group had no perioperative nutrition. Tang et al. [18] analysed the effects of ferric hyaluronate gel on ileus prevention after colorectal surgeries.

### 3.3. Study Outcomes

**Figure 2:**
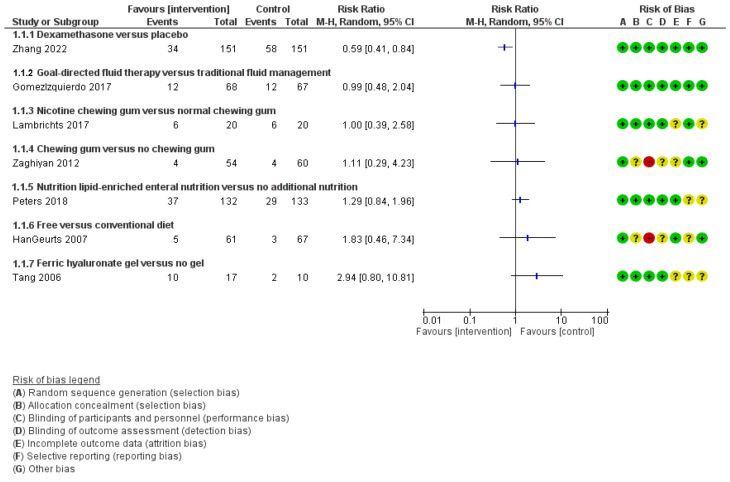
Summary of findings, table of trials investigating interventions and prolonged postoperative ileus after colorectal surgery. RR: relative risk. 95%CI: 95% confidence interval [12,13,14,15,16,17,18].

Our primary outcome was the rate of prolonged ileus. Only one study per intervention was identified. Among these selected studies, different study set ups, procedures and interventions were identified; hence, heterogenicity was high. Consequently, we were not able to perform a conclusive meta-analysis. We assessed the risk of bias within each study. The observed risk ratio was ranked, ranging from 0.59 to 4.41. Zhang et al. [12], in 302 individuals, investigating dexamethasone compared to placebo, obtained a risk ratio of 0.59 [95%CI: 0.41 to 0.84]. The risk ratios, analysis and risk of biases of each study are illustrated in Figure 2.

### 3.4. Risk of Bias (RoB) Outcomes

**Figure 3:**
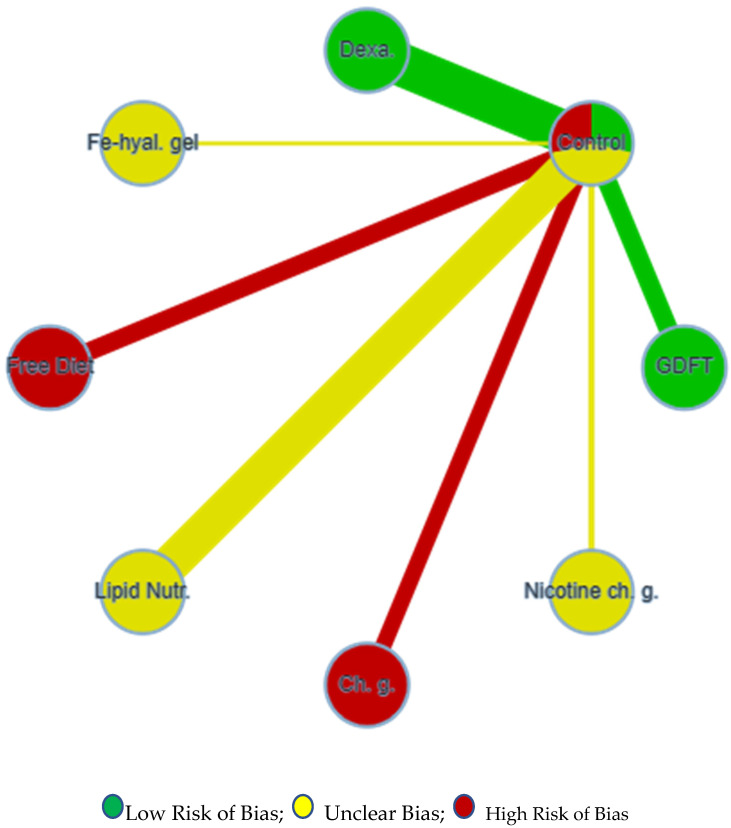
RoB comparison of each intervention to control. Dexa: dexamethasone. Fe-hyal.gel: ferric hyaluronate gel. Lipid Nutr.: lipid nutrition. chg: chewing gum. Nicotine ch.g: nicotine chewing gum. GDFT: goal-directedfluidtherapy.

**Figure 4:**
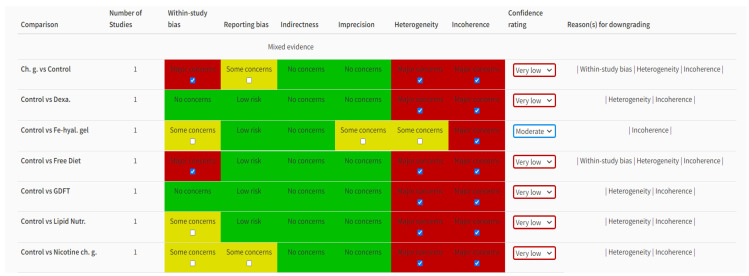
Study analysis of effectiveness. Dexa: dexamethasone. Fe-hyal.gel: ferric hyaluronate gel. Lipid Nutr.: lipid nutrition. chg: chewing gum. Nicotine ch.g: nicotine chewing gum. GDFT: goal-directed fluid therapy. Red: high risk, Yellow: unclear risk, Green: low risk.

After analysing and finding the RoB in each study, we carried out a network meta-analysis for direct and indirect comparisons of the interventions. Since a pairwise (classical) meta-analysis was not considered reliable in this case, a network meta-analysis with a confidence evaluation was performed using the CiNeMA software (version 2.0.0), with interesting and complementary findings to a classical RoB appraisal. A network comparison of each intervention to the control group was encapsulated on an analysis plot from CINeMA (Figure 3). A detailed review of each intervention with an analysis of within-study bias, heterogeneity, incoherence and confidence rating was carried out (Figure 4). This analysis demonstrated that all the studies carried out had very low confidence ratings due to the major concerns of heterogeneity, incoherence and within-study bias except Tang et al. [18], which had a moderate confidence rating. The results with a very low confidence rate pointed out that studies should be more precise with low heterogeneity and concern for incoherence to bring out high-quality data on ileus rate.

## 4. Discussion

### 4.1. Summary of Findings

We identified different knowledge gaps in the field of prolonged ileus and its incidence. Available studies with ileus rates are limited in number (seven studies were included in this systematic review) and highly affected by within-study biases. Due to the high heterogeneity and incoherence of the available studies, a ranking from pairwise comparison and an efficacy analysis of the studies were not possible. The incoherence of the studies also made indirect comparisons unreliable, even if no concern regarding the precision of the outcome measure was identified in most studies.

### 4.2. Interpretation of Results

We have identified seven published randomized controlled trials investigating seven interventions and reporting prolonged postoperative ileus after colorectal surgery. No universal definition was used across the studies. ‘Ileus’ can be defined as a complication after surgeries associated with abdominal distension, functional occlusion, no oral diet tolerance and vomiting with high morbidity or secondary complications [3]. In the excluded studies, ileus was only described as the time until flatus. We did not infer any effect from these studies on ileus since a (typically) 6 to 12 h duration of prolonged time to flatus may only be partially related to the above-mentioned definition of prolonged ileus. Our aim was to focus on prolonged ileus with a patient-centric approach. Ashcroft et al. [3] reported a network meta-analysis on postoperative ileus. They reported outcome data, including the length of hospital stay, time to flatus and time to solid diet tolerance. They found that early feeding was the most efficacious therapeutic intervention to reduce post-operative ileus in patients undergoing colorectal surgery [14]. Our study approach specifically focused on the rate of prolonged ileus, explaining important differences with their findings. In our work, we highlight an important knowledge gap (lack of clinical trials focusing on prolonged ileus rates). This means that more trials are needed to conclude on the efficacy and effectiveness of intervention to prevent prolonged ileus after surgery.

Can we infer, from our findings, more specifically, what kind of trials are required? While discussing the study outcomes and interpreting the data from this systematic review and analysis, we reached some important findings on study designs and the RoB. Due to the variation in the PICO of each study, we have identified the heterogeneity of the studies as of major concern. Heterogeneity, in a (network) meta-analysis, describes a variation when it actually represents variations between researchers as opposed to random variation [11]. In this case, the level of confidence in the point estimate of a relative treatment effect was impacted by the variation in study outcomes. This heterogeneity reduced the confidence rate of the outcome of each intervention, which was expected, but to a much greater extent than the precision did. This observation clearly and specifically draws a conclusion about the significant knowledge gap there is in this area of study. Incoherence was another major finding to interpret from this review. From the CiNeMA analysis, we found that the incoherence between studies is major concern. According to transitivity, two treatments can be compared indirectly using a middle treatment node. Intransitivity would manifest statistically as incoherence; if transitivity is true, the direct and indirect evidence ought to be consistent and coherent. On the other hand, if estimates from the direct and indirect evidence diverge, we can draw the conclusion that transitivity is false and that incoherence exists, which is the case in the current analyses [11]. Incoherence results in indirect comparisons that are not reliable, are non-conclusive and could not have been shown as clearly without performing this confidence evaluation in the meta-analysis using CiNeMA.

### 4.3. Strength and Limitations

This systematic review explored the available studies with a focus on prolonged postoperative ileus prevention. The studies included in the review and meta-analysis were included after multiple database searches and a comprehensive screening, including reference lists. The protocol was registered on PROSPERO before the formal search. The screening, inclusion and data extraction were performed independently by two team members using predefined PICO criteria, which minimised the likelihood of bias in study selection. The main limitation of the review is related to the number of studies matching our PICO. The fact that only seven studies reported data on prolonged ileus rates limited our capacity to compare studies and precluded any meaningful meta-analysis. However, using a consistent approach, we were able to conclude the source of biases by performing a confidence analysis (using CiNeMA).

### 4.4. Perspectives

As major concerns appeared regarding heterogeneity, a clear knowledge gap was identified. Our primary objective was to gather evidence on prolonged postoperative ileus prevention, an important aspect in the postoperative course of patients after colorectal surgery. More than surrogate outcomes, such as time to flatus or length of hospital stay, prolonged postoperative ileus directly impairs quality of life and induces significant morbidity. With the overlap being only partial with these surrogate endpoints, we have specifically identified the fact that more research is required on prolonged ileus rate. Additionally, we observed a high risk of bias in the majority of the studies included is this review. This highlights the need for high-quality randomised controlled trials. Finally, the observed incoherence highlighted the importance of conducting trials on well-defined populations with well-defined and comprehensively defined surgical interventions and using a precise definition of prolonged postoperative ileus.

## 5. Conclusions

This systematic review, with a confidence evaluation in network meta-analysis (CiNeMA), conclusively identified a knowledge gap in the area of prolonged postoperative ileus research and a lack of data on ileus incidence in interventional trials in colorectal surgery. The available studies suffered from a considerable within-study bias, affecting the confidence rates of the study findings. Pairwise (conventional) meta-analyses and ranking of intervention (in network meta-analyses) based on efficacy or effectiveness were not possible due to the paucity of data, heterogeneity and incoherence. Indirect comparisons were not reliable due to this incoherence. These findings are important for planning future studies.

## Figures and Tables

**Table 1:** Characteristics of included studies.

Study	Year	Age: I/C (Median)	Sex: I/C (n)	Proce-Dure	Hemico-Lectomies (n)	Left Hemico-Lectomies/ Anterior (n)	Open/Laparoscopic/Robotic (n)	Intervention	Control
GomezIzquierdo [13]	2017	63/61	M: 31/33 F: 40/24	Colorectal	86	92	Laparoscopic	Goal-directed fluid therapy	Traditional fluid therapy
HanGeurts [14]	2007	63/67	M: 36/25 F:32/35	Colorectal	33	27	Open	Free diet	Conventional diet
Lambrichts [15]	2017	67.5/69	M: 14/6 F: 13/7	Colorectal	14	5	Open: 4 Laparoscopic: 36	Nicotine chewing gum	Normal chewing gum
Peters [17]	2018	69/68	M: 80/52 F: 52/55	Colorectal	94	106	Open: 112 Laparoscopic: 153	Nutrition lipid-enriched enteral nutrition	No perioperative nutrition
Tang [18]	2006	65/67	M: 17/15 F: 9/6	Colorectal	7	10	Open: 29	Ferric hyaluronate gel	No gel
Zaghiyan [16]	2012	42.1/48.8	M: 33/21 F: 34/26	Colorectal	13	15	Open: 44 Laparoscopic: 70	Chewing gum	No gum
Zhang [12]	2022	39.84/42.60	M: 89/62 F: 96/55	Colorectal and others	25	3	Open: 26 Laparoscopic: 68	Dexamethasone	Placebo

M: male. F: female. I: intervention. C: control. Dexa: dexamethasone. Fe-hyal. gel: ferric hyaluronate gel. Lipid Nutr.: lipid nutrition. chg: chewing gum. Nicotine ch.g: nicotine chewing gum. GDFT: goal-directed fluid therapy.

**Figure A1:**
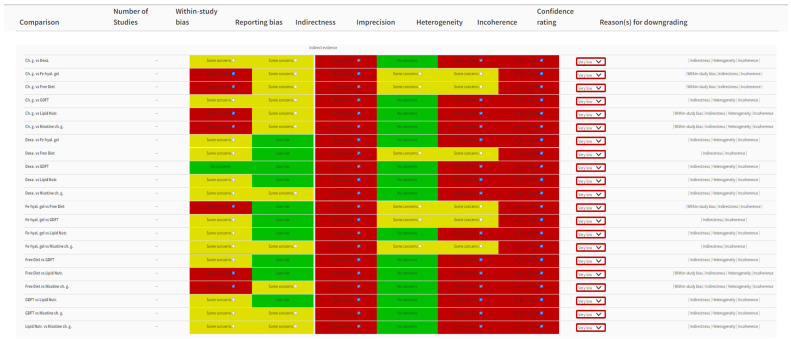
Dexa: dexamethasone. Fe-hyal.gel: ferric hyaluronate gel. Lipid Nutr.: lipid nutrition. chg: chewing gum. Nicotine ch.g: nicotine chewing gum. GDFT: goal-directed fluid therapy.

## Data Availability

All data are included in the main text and Appendix A and Appendix B.

## Appendix A

**Table A1:** Search strategy.

Ovid MEDLINE(R) ALL <1946 to 4 April 2022>	
Search Statement	Results
exp Ileus/	5900
ileus.ab,kf,ti.	12,690
1 or 2	15,480
exp Colon/su [Surgery]	13,689
exp Colorectal Surgery/	4051
exp Rectum/su [Surgery]	12,063
exp Inflammatory Bowel Diseases/su [Surgery]	11,163
Diverticulitis, Colonic/su [Surgery]	1731
Diverticulum, Colon/su [Surgery]	791
Cecal Diseases/su [Surgery]	1464
exp Colorectal Neoplasms/su [Surgery]	47,610
exp Rectal Neoplasms/su [Surgery]	21,676
exp Cecal Neoplasms/su [Surgery]	2054
((colorectal* adj6 surgery) or (colorectal adj6 surgical) or (colorectal adj6 resect*) or (colorectal adj6 laparoscop*) or (colorectal adj6 laparotom*)).ab,kf,ti.	25,019
((colon* adj6 surgery) or (colon* adj6 surgical) or (colon* adj6 resect*) or (colon adj6 laparoscop*) or (colon adj6 laparotom*)).ab,kf,ti.	20,924
((rectum adj6 surgery) or (rectum adj6 surgical) or (rectum adj6 resect*) or (rectum adj6 laparoscop*) or (rectum adj6 laparotom*)).ab,kf,ti.	4669
((rectal adj6 surgery) or (rectal adj6 surgical) or (rectal adj6 resect*) or (rectal adj6 laparoscop*) or (rectal adj6 laparotom*)).ab,kf,ti.	16,876
((“inflammatory bowel” adj6 surgery) or (“inflammatory bowel” adj6 surgical) or (“inflammatory bowel” adj6 resect*) or (“inflammatory bowel” adj6 laparoscop*) or (“inflammatory bowel” adj6 laparotom*)).ab,kf,ti.	1119
((caecal adj6 surgery) or (caecal adj6 surgical) or (caecal adj6 resect*) or (caecal adj6 laparoscop*) or (caecal adj6 laparotom*)).ab,kf,ti.	210
((caecum adj6 surgery) or (caecum adj6 surgical) or (caecum adj6 resect*) or (caecum adj6 laparoscop*) or (caecum adj6 laparotom*)).ab,kf,ti.	146
((cecal adj6 surgery) or (cecal adj6 surgical) or (cecal adj6 resect*) or (cecal adj6 laparoscop*) or (cecal adj6 laparotom*)).ab,kf,ti.	784
((cecum adj6 surgery) or (cecum adj6 surgical) or (cecum adj6 resect*) or (cecum adj6 laparoscop*) or (cecum adj6 laparotom*)).ab,kf,ti.	525
((colitis adj6 surgery) or (colitis adj6 surgical) or (colitis adj6 resect*) or (colitis adj6 laparoscop*) or (colitis adj6 laparotom*)).ab,kf,ti.	2585
((crohn* adj6 surgery) or (crohn* adj6 surgical) or (crohn* adj6 resect*) or (crohn* adj6 laparoscop*) or (crohn* adj6 laparotom*)).ab,kf,ti.	3294
Anal Canal/su [Surgery]	6268
((anal adj6 surgery) or (anal adj6 surgical) or (anal adj6 resect*) or (anal adj6 laparoscop*) or (anal adj6 laparotom*)).ab,kf,ti.	3148
((anus adj6 surgery) or (anus adj6 surgical) or (anus adj6 resect*) or (anus adj6 laparoscop*) or (anus adj6 laparotom*)).ab,kf,ti.	736
((anorectal adj6 surgery) or (anorectal adj6 surgical) or (anorectal adj6 resect*) or (anorectal adj6 laparoscop*) or (anorectal adj6 laparotom*)).ab,kf,ti.	1551
((bowel adj6 surgery) or (bowel adj6 surgical) or (bowel adj6 resect*) or (bowel adj6 laparoscop*) or (bowel adj6 laparotom*)).ab,kf,ti.	17,631
colectomy/ or proctocolectomy, restorative/ or cecostomy/ or colostomy/ or ileostomy/ or proctectomy/	35,635
(“Hartmann’s” or Hartmanns or polypectomy or colectom* or hemicolectomy or ileostomy or proctocolectomy or panproctocolectomy or “pan procto-colectomy” or proctectomy or “anterior resection” or “abdominoperineal resection” or “abdomino-perineal resection” or colostomy or cecostomy).ab,kf,ti.	45,348
(“Ileoanal anastomos*” or “ileo-rectal anastomos*” or “coloanal anastomos*” or “colo-anal anatomos*”).ab,kf,ti.	1438
sigmoidectomy.ab,kf,ti.	1131
(strictureplasty or stricturoplasty).ab,kf,ti.	431
Colonic Polyps/su [Surgery]	2983
((“colonic polyps” adj6 surgery) or (“colonic polyps” adj6 surgical) or (“colonic polyps” adj6 resect*)).ab,kf,ti.	122
or/4-36	156,074
3 and 37	3188
randomized controlled trial.pt.	563,321
controlled clinical trial.pt.	94,786
(randomized or randomised or randomly or groups or placebo or trial).ab,ti.	3,328,560
39 or 40 or 41	3,456,549
38 and 42	821
exp animals/not humans.sh.	4,983,455
43 not 44	795
limit 45 to english language	739
